# Effects of gut microbiome and environment on the development of eczema in Chinese infants

**DOI:** 10.1097/MD.0000000000020327

**Published:** 2020-05-22

**Authors:** Carmen Wing Han Chan, Ting Fan Leung, Kai Chow Choi, Stephen Kwok Wing Tsui, Judy Yuet Wa Chan

**Affiliations:** a The Nethersole School of Nursing; b Department of Paediatrics; c School of Biomedical Sciences, The Chinese University of Hong Kong, Hong Kong.

**Keywords:** eczema, environmental factors, maternal stress, microbiome

## Abstract

**Background:**

Eczema is a relapsing and persistent inflammatory skin disease affecting about one-fifth of children worldwide. As in other developed countries, the prevalence of this chronic disease in Hong Kong is approximately 30%. Moreover, the number of local cases reported has been on a rising trend since 1995. Eczema frequently starts in early infancy. A total of 45% of all cases begin within the first six months of life, 60% during the first year and 85% before the age of 5. The pathophysiology of eczema is multi-factorial and is a complex inter-relationship between skin barrier, genetic predisposition, immunologic development, microbiome, environment, nutrition, and pharmacological and psychological factors.

**Objective:**

To characterize the longitudinal changes of gut microbial profile in early childhood and to examine the association between gut microbiome diversity, environmental factors and the development of eczema in early childhood.

**Method:**

We will conduct a longitudinal cohort study that follows 1250 Hong Kong Chinese infants for 2 years and assess the gut microbiome and other potential environmental factors in the aetiology of eczema. Parents will be asked to provide demographic data, their infant birth data, allergy condition, diet, environmental conditions as well as the data on maternal stress. Stool specimen will be collected for gut microbiome diversity analysis. We will examine newborn infants at enrollment, at 4 months, 1 year and 2 years after birth.

**Expected results:**

This study will evaluate the association between gut microbiome, environmental factors and the development of eczema in Chinese infants. Findings from this study may be used to develop a predictive path model to guide effective health promotion, disease prevention and management.

## 1 Introduction

Eczema is a chronic and relapsing inflammatory skin disease affecting about 20% of children and 3% of adults worldwide.^[1,2]^ Approximately 70% of cases of eczema start in early childhood, under 5, and only about 10% in adulthood.^[3]^ Among the cases of early childhood onset, about 50% develop symptoms within the first year of life.^[1]^ In Hong Kong, the prevalence of eczema is on a rising trend, a 0.12% increase per year since 1995.^[4]^ Summarizing the results of the International Study of Asthma and Allergies in Childhood, Chan et al^[4]^ noted that the prevalence of life-time eczema in children was 30.7%.

The persistence of eczema incurs substantial healthcare costs and impairs the social development of patients because of a poor self-image and lack of self-confidence.^[5]^ It also imposes financial and psychological burdens on the family. Complications of bacterial superinfection and bacteraemia can also be lethal in children.^[6]^ Eczema is always the first step in the atopic march and up to 80% of patients will develop asthma, allergic rhinitis or hay fever later in their lives.^[2,7]^ It is necessary to identify and understand factors in the development of the disease in early childhood which may be pivotal to the onset of the atopic march.

Two theories concerning the component of skin inflammation have been proposed.^[8]^ One holds that the essential deformity dwells in an immunologic aggravation that causes IgE-intervened allergy, with epithelial-boundary brokenness viewed as a result of the neighborhood irritation. The immune reaction may be influenced and driven by environmental stimuli such as allergens resulting from poor air quality or from a diet which may change the gut microbial components. Another speculation recommends that an inherent deformity in the epithelial cells prompts the hindrance brokenness, specifically the microbial substances on the skin. The immunological aspects are considered to be an epiphenomenon.^[8]^ Irritation, lack of sleep and social humiliation due to visible lesions have substantial effects on the psychosocial prosperity of patients and their family members.

The microbial composition is involved in the development of the regulatory T-cell response and thereby plays a key role in immune development in the case of eczema.^[9]^ Inside *in vitro* measures, it has been indicated that the expansion of explicit oligosaccharides during dendritic cell advancement initiates an administrative T-cell reaction of potential advantage in an unfavorably susceptible setting.^[10]^ Moreover, dietary supplementation with specific prebiotic oligosaccharides has been shown to reduce the risk of developing allergies in infants.^[11]^ During early life it therefore seems likely that specific components of the microbial composition can contribute to the normal immune development via multiple direct and indirect pathways, thereby reducing the risk of allergic diseases. Other recently reported birth cohort studies have addressed the relationship of the intestinal microbiome and allergic outcomes, with most suggesting associations.^[12]^

The main contact of mucosal tissues with outside microbiota is vital in the foundation and development of the mucosal, as well as systemic immune systems.^[13]^ In particular, the first year of life is essential to programming the immune system. The development of a barrier function and the immune system are influenced by environmental factors, such as feeding patterns, antibiotic use by the mother during delivery and the use of antibiotics by the neonate.^[14]^ Proper understanding of the protective and programming effects of healthy immune and microbiome development may provide opportunities to reduce the risk of developing eczema. Culture-free studies have given more prominent knowledge into the worldly variances in the bacterial network assorted variety as it creates during a newborn child's first year of life, including dramatic decreases in diversity on antimicrobial administration.^[15]^ However, by approximately 12 months of age, this gut community begins to resemble that of an adult-like microbiome, dominated by the bacterial phyla Firmicutes and Bacteroides and including members of the Proteobacteria, Actinobacteria and Verrucomicrobia, among others.^[15]^ Thus, it has been suggested that this underlying time of colonisation, which coincides with immune response development, represents a vital window during which deviations in colonisation patterns may influence normal immune maturation.^[16]^ Investigators can now take advantage of advances in 16S rRNA gene sequencing and deep sequencing technologies that allow comprehensive detection of microbial communities on a large scale.^[17]^

The prevalence of eczema appears to vary worldwide, with rates ranging from 1% to 20% among children and adults,^[5]^ possibly due to the role of environmental factors in the expression of the disease. Although epidermal gene mutations will have an effect on skin barrier function and provoke an inclination towards immune deviation within the direction of allergic reaction, not all patients with eczema have these mutations. It has been suggested that certain environmental stimuli may be able to trigger both epidermal damage and a Th2 immune reaction regardless of genetic background.^[18]^ In an article published in Science in 2016,^[19]^ it mentioned that “Because microbe-immune interactions appear to play an important role in early-life development of the immune system and when disrupted may result in potentially persistent immune abnormalities, it is worth considering whether early-life exposure to microbes may affect later-life susceptibility to disease. There is emerging evidence to support this hypothesis…”. However, the data on early-life environmental factors and microbiota composition in Hong Kong infants is insufficient. Other environmental factors also play a part. A recent integrative review reports that infant gestational age, delivery mode and feeding type were found to affect gut microbiome composition.^[20]^ Exposure to some antigens seems to increase tolerance and lower the risk of eczema in infants.^[21]^ Dog ownership, the presence of older siblings and probiotic treatment are factors associated with reduced likelihood of allergies. Socio-economic factors, including educational level and parental stress, are also associated with the prevalence of the disease. However, the question of a mechanism linking environmental influences, microbial composition and eczema remains inconclusive,^[22]^ and further scientific investigation is required to confirm or reject.

Both gut microbiome and environmental factors are essentially concerned in the development of eczema, and are also associated with the severity of the disease. In the light of this, we propose to initiate a birth cohort study, examining newborn infants at enrollment, 4 months, 1 year and 2 years after birth. We will access their gut microbiota profile, and measure different environmental factors that may be associated with the disease. This cohort study will help us to understand and pinpoint the gut microbiome and different environmental factors involved in the development and severity of eczema in Chinese children.

## 2 Methods

### 2.1 Study design

This will be a prospective birth cohort study of 1250 Chinese newborns. The subjects will be recruited from Department of Paediatrics in Prince of Wales Hospital in Hong Kong. Subjects’ parents will be asked to provide demographic data, their infant birth data and allergy condition. The cohort will be followed for 2 years, with 3 follow-up measurement points to assess the diet, environment and allergy condition of the infants at the ages of 4 months, 1 year and 2 years. Parental stress will also be assessed. Stool specimens from the newborns will be collected for gut microbiome diversity testing at enrollment, 4 months, 1 year and 2 years of age. It is expected that the whole study will take 36 months to complete.

### 2.2 Subject recruitment

1250 new-born infants will be recruited and followed for two years in the Department of Paediatrics of the Prince of Wales Hospital in Hong Kong. We will recruit full-term infants from the postnatal ward. General population cohort will be included with regardless of family history. The inclusion criteria are in Chinese ethnicity and reside in Hong Kong. The infants with GI disorders after birth, received intravenous injection of antibiotics, admitted to neonatal ICU, whose mothers have fever or an infection or are currently taking antibiotics will be excluded.

### 2.3 Ethical considerations

This study was registered on the ISRCTN Registry with reference number ISRCTN44080054. The study protocol was approved by the Joint Chinese University of Hong Kong – New Territories East Cluster Clinical Research Ethics Committee with reference number 2016.321. Any modifications to the protocol that may have an impact on the execution of the study will be reported to the committee. Potential subjects’ parents will be given detailed information about the study and their rights to participate. Written informed consent will be obtained from the subjects’ parents prior to data collection.

### 2.4 Sample size estimation

1250 new-born infants will be recruited. The hospital averages of 5000 deliveries per year. According to our anecdotal prevalence data, about 30% (∼375 cases) of the infants will develop eczema in the first 2 years of life. It is estimated that an accumulation of 375 event cases would be sufficient to detect a hazard ratio of a standardised normally distributed continuous risk factor and a binary risk factor as small as 1.16 and 1.94 respectively, with 80% power at a 1-sided 2.5% level of significance, on the basis of the very mild assumption that the prevalence of underlying binary risk factors is not too extreme, that is, within 5% to 95%. Moreover, this sample size would also be adequate to detect a binary risk factor with a risk ratio (RR) as small as 1.42 to 2.00 and a normally distributed continuous risk factor with a RR as small as 1.17 to 1.44 at 80% power and 1-sided 2.5% level of significance, provided the prevalence of the underlying binary risk factor was within 5% to 95% and the probability of eczema at the mean value of the continuous risk factor was also within 5% to 95%. This study also aims to examine the mediating role of microbiome diversity on the relationships between environmental and lifestyle risk factors and development of eczema using path analysis. A commonly used rule of thumb^[23]^ suggests that a sample size of at least 100 and preferable ≥ 200 is required for conducting path analysis. In consideration of both statistical and financial criteria, a random subsample of totally 150 children of the cohort will be undergone gut microbiome examination of their stool specimens collected.

### 2.5 Data collection

#### 2.5.1 Demographic characteristics

Demographic data will be collected at enrolment by self-report. This information includes: gender, body weight and body length of the infant, gestational age, mode of delivery, feeding practice (breast or formula feeding), emollient use, parents’ education level and household income.

#### 2.5.2 Allergy condition

The infant's allergy condition will be assessed by the modified parent proxy version of the Comprehensive Early Childhood Allergy Questionnaire developed by Minasyan and colleagues. Data will be collected by a research assistant at enrollment, and at the age of 4 months, 1 year and 2 years.

Comprehensive Early Childhood Allergy Questionnaire is designed to detect eczema, asthma and IgE-mediated food allergies in children aged from 1 to 5. It assesses allergic diseases, family history of allergy, parental socio-economic status, parental smoking and child's feeding patterns. It has been validated with good psychometric properties and has been translated and modified to reflect the Chinese situation under the standard procedure before being used in this study. Some questions, such as those concerned with the history of infection, use of antibiotic, pet ownership, air quality, number of siblings and mode of delivery, have been added to the questionnaire. The modified questionnaire has been reviewed by an expert panel of paediatricians, geneticists, paediatric nurses, academics, medical scientists and parents of children with eczema. The feasibility, appropriateness and acceptability of the questionnaire are confirmed by five proxy parents of eczematous children, taking an average of 7 minutes to answer the questionnaire in full.

#### 2.5.3 Diet and use of supplements

The subjects’ dietary factors will be assessed by a parent proxy dietary practice questionnaire, adopted from the Chinese version of the eating-habit module of the Behavioural Risk Factor Surveillance System, Hong Kong Centre for Health Protection. The information will be collected from parents when the children are at the age of 4 months, 1 year and 2 years. Information on the mother's diet and use of supplements during pregnancy will be collected after the birth by means of the same questionnaire.

#### 2.5.4 Maternal stress

Maternal stress will be assessed at 4 time points: at enrollment, at 4 months, 1 year and 2 years. Mothers will be asked to complete the 10-item perceived stress scale to assess their own stress levels. There are 10 items, measured on a five-point Likert scale, from 0 (never) to 4 (very often). The Chinese version has been reported to have good validity and internal consistency.^[24]^

### 2.6 Gut microbiome metagenomics analysis

A total of 150 infants who confirmed to have eczema based on the assessment by the Scoring Atopic Dermatitis (SCORAD) index at 2-year will be selected for microbiome diversity profiling. A similar infant microbiome study has demonstrated that a sample size of 100 is enough for the detection of significant changes in the gut community composition.^[25]^ Infants will be followed for 2 years for development of eczema and other allergic diseases such as recurrent wheeze, food allergy and rhinitis, and the stored stool specimens of a stratified random subsample of 150 infants will be selected for gut microbiome analysis on the basis of severity of eczema as assessed by the SCORAD index at 2-year. Fifteen children in each of the 10 strata based on the deciles of the SCORAD index will be randomly selected using computerized randomization. Stool samples from all newborns will be collected from the napkin and suspended in 0.6 mL lysis buffer (TIANGEN Biotech, Beijing, China). The colonisation and development of the gut microbiome will be followed at 10 days post-birth, 4 months, 1 year and 2 years (modified from Storrø et al^[22]^). Stool samples will be stored at -80°C before analysis. Total microbial DNA will be extracted from the stool using the TIANamp stool DNA kit. The quantity and quality of the total microbial DNA will be verified by the NanoDrop 1000 spectrophotometer (Thermal Scientific, Texas, USA) and Bioanalyser 2100 (Agilent Technologies, California, USA) respectively. A total of 600 metagenomic libraries from 4-time points (at enrollment, 4 months, 1 year and 2 years) of 150 infants will be constructed using the PCR primers flanking the V3-V4 region of the 16S ribosomal RNA gene. All PCR will be performed in triplicate to minimise the effect of amplification bias. Following PCR using the high-fidelity enzyme, amplicons with different barcodes will be analysed using the Bioanalyser 2100 and Qubit spectrophotometer. Equimolar amounts of amplicon libraries (∼80 libraries per run) will be pooled for next-generation sequencing using the MiSeq platform (Illumina, California, USA).

### 2.7 Data processing analysis

The metagenomic data set will be analysed using the open source bioinformatics tool Mothur. Briefly, cleaned sequence reads will be trimmed and aligned to the bacterial-subset SILVA, followed by filtering to remove vertical gaps. The reads are then screened for potential chimeras using the uchime method and finally classified using the Ribosomal Database Project's naïve Bayesian classifier training set for Mothur. Differentially abundant bacterial taxa, PcoA loadings, species richness (Sobs), Shannon's diversity (H’) and evenness (EH) and Simpson's diversity will be calculated using the Mothur workflow.

Data will be suitably transformed, if applicable, before being subjected to inferential analyses. In particular, the frequency data of relative abundance of the differentially abundant bacterial taxa identified will be arc-sine square root-transformed. A mixed-effects model will be used to identify environment and lifestyle risk factors associated with the development of eczema by using a log-binomial link function for estimating the relative risks of the factors identified based on the whole cohort. If a more exact time of eczema development can be recorded, Cox regression with possibly time-varying covariates will be used instead to examine environment and lifestyle risk factors associated with the development of eczema. A mixed-effects model will also be used to examine the longitudinal association of gut microbiome diversity in the development of eczema by using the sub-sample with microbiome data. Furthermore, path analysis will be performed to examine whether the mediating role of microbiome diversity in the relationships between environmental risk factors and the development of eczema based on the random sub-sample. The significance of the mediating effects will be determined by bias-corrected bootstrapping method with 10,000 replications. The bootstrapping will be performed using Matlab 7.0 (The Mathworks, Inc). Except as specified above, all other statistical analyses will be performed using SAS release 9.4 (SAS Institute, Cary, NC).

## 3 Discussion

Eczema is a common chronic inflammatory skin disorder that is regarded as a typical multifactorial disease. It affects up to 230 millions of people globally.^[26]^ Onset is usually in the first few weeks and months of life and, while most affected people enter spontaneous remission, the dermatitis may persist into adult life. The etiology is complex and the disease is caused by a combination of immune dysregulation, genetic susceptibility, environmental factors and impaired barrier function.^[27,28]^

There is a substantial economic cost not only to the family of the person with eczema but also to health services.^[29]^ In 1 study, a published work review was conducted to estimate the economic burden of pediatric eczema patients in the Asia–Pacific region and showed that the annual direct cost of eczema ranged from USD199 to USD1,250.^[30]^ Although there is currently no cure for eczema, a wide range of treatments are used to control the symptoms. One such approach is the change of gut microbiome through the diet. Colonization of the infant gastrointestinal tract by microbes is an essential process in our life cycle since microbiota–host interactions have an important influence on human health and disease.^[15]^ Infancy is a critical period for the colonization of the gut microbiome, which affects host immune development. Following birth, the human intestine is rapidly colonized by an array of microbes and factors known to influence colonization include gestational age, mode of delivery (vaginal birth vs assisted delivery), diet (breast milk vs formula), sanitation, and antibiotic treatment^[31,32]^ Therefore, perturbation of the infant gut microbiome can shape the development of the immune system and link to the risk of allergic disease including atopic dermatitis.^[31,33]^ People with eczema have different bacteria in their gut compared to people without eczema, and sometimes they have inflammation in their gut.^[34,35]^ Several factors can affect colonization of the gut microbiome, and the feeding type is the greatest factor on the colonization of the gut microbiome in early life. It has been suggested that eczema symptoms may be treated by changing the mix of gut bacteria or by reducing inflammation in the gut. Probiotics, which are live micro-organisms taken by mouth, such as the Lactobacillus bacteria found in unpasteurised milk and yoghurt, might achieve this.^[36,37]^ However, studies on the complex relationship between the gut microbiome and atopic dermatitis according to feeding type in early life are limited. In this study, the feeding style, the use of antibiotics, probiotics and other supplements will be investigated.

Another vital part in eczema development is the environmental issue. Environmental allergen levels are probably the most important determinant of whether or not allergy of genetically susceptible people happens. Increased exposure to sensitizing allergens and reduced stimulation of the immune system during critical periods of development are seen as the main factors driving the rising prevalence of allergic diseases. The IgE-mediated antigen presentation of allergens has been recognized as a key event in the pathogenesis of eczema.^[38,39]^ The main principles of eczema management are allergen and irritant avoidance, emollient use and topical or systemic anti-inflammatory treatment.^[40]^ The most common allergens leading to allergy in childhood are house-dust mite, grass pollen, etc. Allergens causing type-1 hypersensitivity reaction should clearly be avoided and there is some proof that reducing dust-mite exposure in the subset of susceptible patients improves eczema management.^[41]^ In this study, the factors such as pet at home, air quality, day care condition, family history will be examined.

Review articles suggested that prenatal maternal distress might also increase the risk of allergic disease in children.^[42,43]^ Recently, a birth cohort study showed that prenatal maternal psychological distress also increases the risk of eczema.^[44]^ This was supported by 2 more recent birth cohort studies that showed that when mothers experienced stressful life events during pregnancy, their children had an increased risk of eczema.^[45,46]^ Given the significant medical and psychological burden of eczema, further investigation into the association between maternal distress and subsequent eczema in offspring is warranted.

Currently, there is no cure for eczema. Proper treatment and good skin care can often control or minimize symptoms. However, prevention is the best way to manage eczema. For that reason, it is important to identify and avoid symptom triggers, such as allergens or improper diet. In long term, improving the immunity and reducing inflammation are beneficial to the affected people, especially the infants. Therefore, the aims of this study is to identify the association of early-life gut microbiome, environmental, lifestyle and maternal stress risk factors in the development of eczema. This study is significant in that it will provide evidence on the factors associated with early development of eczema. The results of this study may lead to other descriptive and interventional studies that will evaluate different approaches to prevent, treat and manage the disease of eczema.

This study started in September 2016 and is under on-going status at the present time.

## Author contributions

Carmen Chan is responsible for study design.

Stephen Tsui provide suggestions on microbiome analysis.

TF Leung provides suggestions on recruitment of participants.

KC Choi is responsible for data analysis.

Judy Chan is responsible for data collection and experimental work.

All of the authors contributed to revisions of the manuscript and its final approval.

## References

[1] Thomsen SF Atopic dermatitis: natural history, diagnosis, and treatment. ISRN Allergy 2014;2014:354250. 25006501 10.1155/2014/354250 PMC4004110

[2] Plötz SG Wiesender M Todorova A What is new in atopic dermatitis/eczema? Expert Opin Emerg Drugs 2014;19:441–58. 25286776 10.1517/14728214.2014.953927

[3] Williams HC Clinical practice. Atopic dermatitis. N Engl J Med 2005;352:2314–24. 15930422 10.1056/NEJMcp042803

[4] Chan YT Ho HK Lai CK Hong Kong Allergy Alliance. Allergy in Hong Kong: an unmet need in service provision and training. Hong Kong Med J 2015;21:52–60. 25554794 10.12809/hkmj144410

[5] Deckers I McLean S Linssen S Investigating international time trends in the incidence and prevalence of atopic eczema 1990-2010: a systematic review of epidemiological studies. PloS One 2012;7:e39803. 22808063 10.1371/journal.pone.0039803 PMC3394782

[6] Mrabet-Dahbi S Dalpke AH Niebuhr M The Toll-like receptor 2 R753Q mutation modifies cytokine production and Toll-like receptor expression in atopic dermatitis. J Allergy Clin Immunol 2008;121:1013–9. 18234309 10.1016/j.jaci.2007.11.029

[7] van der Hulst AE Klip H Brand PL Risk of developing asthma in young children with atopic eczema: a systematic review. J Allergy Clin Immunol 2007;120:565–9. 17655920 10.1016/j.jaci.2007.05.042

[8] Bieber T Atopic dermatitis. N Engl J Med 2008;358:1483–94. 18385500 10.1056/NEJMra074081

[9] Round JL Mazmanian SK Inducible Foxp3+ regulatory T-cell development by a commensal bacterium of the intestinal microbiota. Proc Natl Acad Sci USA 2010;107:12204–9. 20566854 10.1073/pnas.0909122107 PMC2901479

[10] Lehmann S Hiller J van Bergenhenegouwen J In vitro evidence for immune-modulatory properties of non-digestible oligosaccharides: direct effect on human monocyte derived dendritic cells. PLoS One 2015;10:e0132304. 26148091 10.1371/journal.pone.0132304 PMC4493044

[11] Boyle RJ Tang ML Chiang WC Prebiotic-supplemented partially hydrolysed cow's milk formula for the prevention of eczema in high-risk infants: a randomized controlled trial. Allergy 2016;71:701–10. 27111273 10.1111/all.12848 PMC4996326

[12] Wegienka G Zoratti E Johnson CC The role of the early-life environment in the development of allergic disease. Immunol Allergy Clin North Am 2015;35:1–7. 25459574 10.1016/j.iac.2014.09.002 PMC4427897

[13] Van’t Land B Schijf MA Martin R Influencing mucosal homeostasis and immune responsiveness: the impact of nutrition and pharmaceuticals. Eur J Pharmacol 2011;668: Suppl. 1: S101–7. 21810416 10.1016/j.ejphar.2011.05.082

[14] Ahmadizar F Vijverberg SJH Arets HGM Early life antibiotic use and the risk of asthma and asthma exacerbations in children. Pediatr Allergy Immunol 2017;28:430–7. 28423467 10.1111/pai.12725

[15] Palmer C Bik EM Digiulio DB Development of the human infant intestinal microbiota. PLoS Biol 2007;5:e177. 17594176 10.1371/journal.pbio.0050177 PMC1896187

[16] Penders J Thijs C van den Brandt PA Gut microbiota composition and development of atopic manifestations in infancy: the KOALA Birth Cohort Study. Gut 2007;56:661–7. 17047098 10.1136/gut.2006.100164 PMC1942165

[17] Poretsky R Rodriguez-R LM Luo C Konstantinidis strengths and limitations of 16S rRNA gene amplicon sequencing in revealing temporal microbial community dynamics. PLoS ONE 2014;9:e93827. 24714158 10.1371/journal.pone.0093827 PMC3979728

[18] Eyerich K Novak N Immunology of atopic eczema: overcoming the Th1/Th2 paradigm. Allergy 2013;68:974–82. 23889510 10.1111/all.12184

[19] Gensollen T Iyer SS Kasper DL How colonization by microbiota in early life shapes the immune system. Science 2016;352:539–44. 27126036 10.1126/science.aad9378 PMC5050524

[20] Chan CWH Wong R Law PTW A review of external factors contributing to altered gut microbiota for childhood eczema. Sixth Pan-Pacific Nursing Conference 2016.

[21] Flohr C Mann J New insights into the epidemiology of childhood atopic dermatitis. Allergy 2014;69:3–16. 24417229 10.1111/all.12270

[22] Storrø O Øien T Langsrud Ø Temporal variations in early gut microbial colonization are associated with allergen-specific immunoglobulin E but not atopic eczema at 2 years of age. Clin Exp Allergy 2011;41:1545–54. 21749499 10.1111/j.1365-2222.2011.03817.x

[23] Wolf EJ Harrington KM Clark SL Miller MW Sample size requirements for structural equation models an evaluation of power, bias, and solution propriety. Educ Psychol Meas 2013;73:913-934 10.1177/0013164413495237 PMC4334479 25705052

[24] Lu W Bian Q Wang W Chinese version of the Perceived Stress Scale-10: A psychometric study in Chinese university students. PLoS One 2017;12:e0189543. 29252989 10.1371/journal.pone.0189543 PMC5734731

[25] Madan JC Hoen AG Lundgren SN Association of cesarean delivery and formula supplementation with the intestinal microbiome of 6-week-old infants. JAMA Pediatr 2016;11:1–8. 10.1001/jamapediatrics.2015.3732 PMC4783194 26752321

[26] Rodríguez JM Murphy K Stanton C The composition of the gut microbiota throughout life, with an emphasis on early life. Microb Ecol Health Dis 2015;26:26050. 25651996 10.3402/mehd.v26.26050 PMC4315782

[27] Weidinger S Novak N Atopic dermatitis. Lancet 2016;387:1109–22. 26377142 10.1016/S0140-6736(15)00149-X

[28] Nutten S Atopic dermatitis: global epidemiology and risk factors. Ann Nutr Metab 2015;66: suppl 1: 8–16. 25925336 10.1159/000370220

[29] Tsai TF Rajagopalan M Chu CY Burden of atopic dermatitis in Asia. J Dermatol 2019;46:825–34. 31436343 10.1111/1346-8138.15048

[30] Lee BW Detzel PR Treatment of childhood atopic dermatitis and economic burden of illness in Asia Pacific countries. Ann Nutr Metab 2015;66: suppl 1: 18–24. 10.1159/000370221 25925337

[31] Marques TM Wall R Ross RP Programming infant gut microbiota: influence of dietary and environmental factors. Curr Opin Biotechnol 2010;21:149–56. 20434324 10.1016/j.copbio.2010.03.020

[32] Adlerberth I Wold AE Establishment of the gut microbiota in Western infants. Acta Paediatr 2009;98:229–38. 19143664 10.1111/j.1651-2227.2008.01060.x

[33] Avershina E Storrø O Øien T Major faecal microbiota shifts in composition and diversity with age in a geographically restricted cohort of mothers and their children. FEMS Microbiol Ecol 2014;87:280–90. 24112053 10.1111/1574-6941.12223

[34] Tang MF Sy HY Kwok JS Eczema susceptibility and composition of faecal microbiota at 4 weeks of age: a pilot study in Chinese infants. Br J Dermatol 2016;174:898–900. 26408944 10.1111/bjd.14205

[35] Salameh M Burney Z Mhaimeed N The role of gut microbiota in atopic asthma and allergy, implications in the understanding of disease pathogenesis. Scand J Immunol 2020;91:e12855. 31793015 10.1111/sji.12855

[36] Makrgeorgou A Leonardi-Bee J Bath-Hextall FJ Cochrane Skin Group. Probiotics for treating eczema. Cochrane Database Syst Rev 2018;2018:CD006135. 10.1002/14651858.CD006135.pub3 PMC6517242 30480774

[37] Huang R Ning H Shen M Probiotics for the treatment of atopic dermatitis in children: a systematic review and meta-analysis of randomized controlled trials. Front Cell Infect Microbiol 2017;7:392. 28932705 10.3389/fcimb.2017.00392 PMC5592329

[38] Gilles S Akdis C Lauener R The role of environmental factors in allergy: a critical reappraisal. Exp Dermatol 2018;27:1193–200. 30099779 10.1111/exd.13769

[39] Matsumoto K Saito H Epicutaneous immunity and onset of allergic diseases - per-“eczema”tous sensitization drives the allergy march. Allergol Int 2013;62:291–6. 23974875 10.2332/allergolint.13-RAI-0603

[40] Brown SJ Atopic eczema. Clin Med 2016;16:66–9. 10.7861/clinmedicine.16-1-66 PMC4954337 26833520

[41] Nankervis H Pynn EV Boyle RJ House dust mite reduction and avoidance measures for treating eczema. Cochrane Database Syst Rev 2015;1:CD008426. 25598014 10.1002/14651858.CD008426.pub2 PMC8407038

[42] Kozyrskyj AL Bahreinian S Azad MB Early life exposures: impact on asthma and allergic disease. Curr Opin Allergy Clin Immunol 2011;11:400–6. 21772139 10.1097/ACI.0b013e328349b166

[43] Yamamoto N Nagano J Parental stress and the onset and course of childhood asthma. Biopsychosoc Med 2015;9:7. 25741380 10.1186/s13030-015-0034-4 PMC4349604

[44] Wen HJ Wang YJ Lin YC Prediction of atopic dermatitis in 2-yr-old children by cord blood IgE, genetic polymorphisms in cytokine genes, and maternal mentality during pregnancy. Pediatr Allergy Immunol 2011;22:695–703. 21539617 10.1111/j.1399-3038.2011.01177.x

[45] Hartwig IR Sly PD Schmidt LA Prenatal adverse life events increase the risk for atopic diseases in children, which is enhanced in the absence of a maternal atopic predisposition. J Allergy Clin Immunol 2014;134:160–9. 25117802 10.1016/j.jaci.2014.01.033

[46] De Marco R Pesce G Girardi P Foetal exposure to maternal stressful events increases the risk of having asthma and atopic diseases in childhood. Pediatr Allergy Immunol 2012;23:724–9. 22957808 10.1111/j.1399-3038.2012.01346.x

